# Cervical cancer screening outcomes in public health facilities in three states in Nigeria

**DOI:** 10.1186/s12889-023-16539-1

**Published:** 2023-09-01

**Authors:** Olufunmilayo Lawson, Lola Ameyan, Zainab Tukur, Sophia Dunu, Matilda Kerry, Oluwapelumi Ololade Okuyemi, Zainab Yusuf, Olufunke Fasawe, Owens Wiwa, Katharine Schilling Hebert, Jessica Trenc Joseph, Uchechukwu Emmanuel Nwokwu, Okpikpi Okpako, Christopher Ifeanyi Chime

**Affiliations:** 1 Clinton Health Access Initiative, Abuja, Nigeria; 2 Clinton Health Access Initiative, Boston, MA USA; 3 Federal Ministry of Health, Abuja, Nigeria; 4 Institute of Human Virology, Abuja, Nigeria

**Keywords:** Cervical cancer, Screening, Treatment, Precancerous lesions, Linkage, Referral, Public facilities

## Abstract

**Background:**

Cervical cancer continues to generate a significant burden of disease and death in low- and middle-income countries (LMICs). Lack of awareness and poor access to early screening and pre-cancer treatment contribute to the high mortality. We describe here cervical cancer screening outcomes in public health facilities in three states in Nigeria.

**Methods:**

We conducted an observational study in 177 government health facilities in Lagos, Kaduna, and Rivers State, Nigeria from January to December 2021, in which we reviewed programmatic data collected through the newly introduced Cervical Cancer Prevention Program. Women who received screening and provided consent were enrolled into the study. Data were extracted from registers in the health facilities using SurveyCTO and descriptive statistical analysis was conducted using StataSE 15 (StataCorp, College Station, TX, USA).

**Results:**

Eighty-three thousand, five hundred ninety-three women were included in the analysis including 6,043 (7%) WLHIV. 67,371 (81%) received VIA as their primary screening while 16,173 (19%) received HPV DNA testing, with 49 (< 1%) receiving both at the same time. VIA positivity was 7% for WLHIV and 3% for general population, while HPV prevalence was 16% for WLHIV and 8% for general population. Following a positive HPV result, 21% of women referred, completed triage examination. 96% of women identified with precancerous lesions, received treatment. 44% of women with suspected cancer were successfully referred to an oncology center for advanced treatment. Following treatment with thermal ablation, seven adverse events were reported.

**Conclusions:**

The Program has successfully increased women’s access to screening and treatment of precancerous lesions. Almost all women who were eligible for pre-cancerous lesion treatment received it, often on the same day when screened using VIA. However, for women referred for a triage exam or due to suspected cancer, many did not complete their referral visits. More effort is required to ensure HPV positive women and women with suspected cancer are adequately linked to care to further reduce morbidity and mortality associated with cervical cancer in Nigeria. Implementation studies should be conducted to provide insights to improve the utilization of the existing centralized and point of care (POC) platforms to facilitate same day results, and to improve triage and treatment rates.

## Background

Cervical cancer affects more than half a million women each year and disproportionately impacts women in LMICs, where nearly nine in ten deaths due to cervical cancer occur [1]. It is the fourth most common cause of cancer deaths in women globally and the top cause of cancer deaths in women in eastern, western, middle, and southern Africa [1]. Nigeria contributes significantly to the global burden of cervical cancer. The incidence of cervical cancer in Nigeria is about 18.4 per 100,000 women, with an estimated 12,075 women diagnosed every year [2]. This makes cervical cancer the second leading cause of female cancer deaths in Nigeria. The inequitable distribution of cervical cancer cases and deaths is directly linked to disparities in access to secondary prevention: countries with robust screening programs have cut cervical cancer deaths by 50% [3].

Cervical cancer is caused by persistent genital high-risk human papillomavirus (HPV) infection [4]. While most HPV infection is usually cleared without development of disease [5], some women will develop precancerous lesions, which if left untreated, can then progress to invasive cancer. Women living with HIV (WLHIV) are more likely to have high-risk HPV infections and are at greater risk that these infections will progress to cervical cancer [6]. HIV care and treatment programs offer a critical prevention opportunity to reach WLHIV with screening and treatment for pre-cancerous lesions.

The World Health Organization (WHO) has launched a Call to Action for the elimination of cervical cancer [7]. In response to this call, key stakeholders, including donors and national governments, are prioritizing cervical cancer in their public health plans and budgets. Elimination relies on the effective scale-up of both HPV vaccination (primary prevention) and screening and treatment for precancerous lesions (secondary prevention). Even as countries introduce the HPV vaccine, screening at regular intervals is still important for both vaccinated and unvaccinated women. Screening coverage needs to reach 70% of eligible women to result in a decrease in the incidence of cervical cancer [8].

Many LMICs rely on visual inspection with acetic acid (VIA) as the primary screening method due to its low cost, minimal material requirements, and the ability to make a diagnosis without the need to transport specimens, use laboratory equipment, or utilize pathologists. The efficacy of VIA, however, is highly dependent on provider training and experience, and can lack both intra- and inter-observer reliability [9, 10]. HPV DNA testing is increasingly being used for cervical cancer screening because it can be collected easily by either the provider or the patient, and it provides more objective results with better sensitivity than VIA [11] and is recommended over VIA (when feasible) by the WHO [12]. Furthermore, to effectively prevent cervical cancer, screening needs to be tied to prompt treatment for pre-cancerous lesions with cryotherapy and Loop Electrosurgical Excision Procedure (LEEP) currently being the preferred and available treatment methods to treat cervical precancerous lesions in many LMICs.

The Nigerian Government has expressed a keen interest in identifying affordable, effective tools to bring cervical cancer screening and treatment to scale. While the Nigerian government understands the shortcomings of VIA, similar to Governments of other LMICs, it also finds HPV testing unaffordable. Additionally, cryotherapy is the most widely used treatment method in country. The reliance of current cryotherapy tools on medical gas supply chain prevents widely available treatment, due to frequent gas stock-outs and significant ongoing operating costs. Gasless treatment tools such as thermal ablation devices can make point-of-care treatment readily available, easing logistics and reducing the risk of loss-to-follow-up. Thermal ablation has comparable effectiveness to cryotherapy for treatment of pre-cancerous lesions, the devices are considerably easier to use and manage and the procedure is safe, with minimal side effects and adverse events, and no measurable impact on fertility [13–15]. As a result, many LMICs have already started using these Stringent Regulatory Authority approved devices at a small scale.

The lack of affordable, practical methods of screening and treatment for cervical cancer puts the Nigerian government in a difficult position as they seek to expand access to high-quality secondary prevention. As there is currently limited evidence from LMICs on implementation of preparing for and rolling out cervical cancer prevention services at scale, especially using new technologies including HPV testing and thermal ablation, there is need to describe the implementation and integration of practical routine cervical cancer screening and treatment services including the introduction of HPV DNA testing and thermal ablation, into the public health system.

## Methods

### Summary of the cervical cancer secondary prevention program

With support from Unitaid, the Clinton Health Access Initiative (CHAI), in partnership with the Nigerian Federal Ministry of Health (MoH), introduced a cervical cancer screening and treatment program in three states: Kaduna, Lagos and Rivers. The goal was to develop and scale up optimal screening and treatment models in country by integrating services across key entry points such anti-retroviral clinics, family planning clinics, antenatal clinics, or labor/delivery units. The program aimed to screen 170,000 women including approximately 34,500 WLHIV (20%) across 177 government health facilities between January 2021 and December 2022. Women could be screened through VIA or HPV Test as a primary screen with 17,265 tests i.e., 30% of total HPV tests allocated to WLHIV, and the remaining 39,960 tests (70%) allocated to general population women. The program also targeted WLHIV between 25 and 49 years old, and women from the general population between 30 and 49 years old.

A national training curriculum was developed in collaboration with the Federal Ministry of Health, with senior medical doctors across the country identified and trained to be National Cervical Cancer Trainers. In each state, senior medical doctors were identified and trained by national trainers as state trainers, and cascade trainings conducted to other healthcare workers in the states to provide high quality cervical cancer screening and treatment including counselling. Following the launch of screening and treatment at the program sites, a mentoring approach was also instituted, with the state trainers providing clinical mentoring to healthcare workers (HCWs) periodically over an eight-week period. The purpose of this was to reinforce learnings from the HCW trainings and to monitor and strengthen the quality-of-service provision among HCWs.

To raise disease awareness and drive uptake of cervical cancer screening services, the program also implemented robust demand generation strategies including collaborating with established Civil Society Organizations (CSOs) and leveraging their trust-based relationships with the community to reiterate the importance of cervical cancer screening, conducting community outreaches as well as facility in-reach programs, and training of over 170 community mobilizers on demand generation in order to reach mass numbers of women with key messages on cervical cancer screening.

**Fig. 1:**
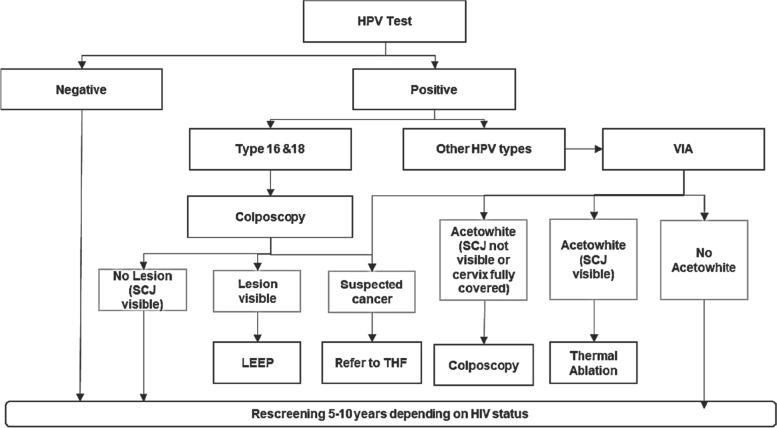
Cervical cancer screening algorithm

All health facilities within the program offered screening with HPV DNA testing and/or VIA, on-site treatment for eligible women using thermal ablation, treatment, or referral for LEEP for more severe/ larger lesions, and referral for management of suspected cancer. 43 of the 177 sites had LEEP available on site while 6 of the 177 sites were tertiary institutions with oncology centers. HPV testing occurred on both GeneXpert and Roche platforms, with on-site testing at health facilities equipped with GeneXpert devices, and sample transportation to centralized labs with Roche platforms in country. Follow-up for women who underwent cervical cancer screening was determined based on their screening results per the national cervical cancer prevention training algorithm (Fig. 1). Per the algorithm, all women identified as HPV-positive are to be referred for a triage examination using VIA or colposcopy. Women who screen positive with VIA where pre-cancerous lesions are evident, either upon initial screening or during a triage examination, are to be treated using thermal ablation or LEEP. Women with suspected cancer are to be referred to tertiary facilities for further management.

As part of the program, a robust patient tracking system was implemented to monitor patient progress through the continuum of care. National data tools including screening and treatment forms, screening and treatment register, referral card and referral tracker were developed in collaboration with the FMOH to standardize data collection for cervical cancer secondary prevention. Furthermore, 354 patient navigators and focal mentees were trained on patient tracking and referral pathways across the three states. These HCWs were trained to contact women via phone calls to provide screening results, and to remind women to attend their referral visit or to visit facilities for further treatment and care. They were also trained on the use of a referral tracker to monitor referral completion.

### Study design and participants

We conducted an observational descriptive study in which we collected routine programmatic data on women accessing screening for cervical cancer at the cervical cancer secondary prevention program sites from January to December 2021. Using this data, we conducted secondary analyses to monitor the status of screening and treatment of precancerous cervical lesions and understand program implementation over time. Study outcomes included the number of women screened for cervical cancer, the number of screen-positive women eligible for treatment who were treated, the number of screen-positive women referred for suspected cancer who attended the referral visit, and the number of women suffering adverse events after treatment of precancerous lesions with thermal ablation.

Women were eligible for study inclusion if screened with VIA or HPV DNA testing, living with HIV and aged 25 to 49 years, or if HIV negative or of unknown HIV status and aged 30 to 49 years. If a woman did not provide informed consent, she still received cervical cancer screening, but her information was not collected as part of the study.

### Data collection

Data were extracted from facility level paper-based tools completed by healthcare providers, namely the Cervical Cancer Screening and Treatment Register, facility Referral Tracker, and facility adverse event tracker logbooks. The patient level data collected included age, HIV status, cervical cancer screening history, method of cervical cancer screening, screening results, treatment received if any, referral, referral completion and results of any follow up visit. Trained study data collectors visited each focal facility approximately once a month, reviewed the facility level paper-based tools and abstracted the targeted data using an electronic data collection tool, SurveyCTO, on a mobile device. The abstracted data was then uploaded and stored on a password-protected, secure cloud-based server.

### Sample size

All patients receiving cervical cancer screening using VIA or HPV DNA testing, treatment of precancerous lesions, or referral for suspected cancer, within the target age group, study timeframe, and who provided informed consent were included in this study, therefore the sample size was dependent on service uptake.

### Data analysis

Data analysis was descriptive in nature. Numbers and percentages for categorical variables are presented. For continuous variables the mean, standard deviation, median, range, and number of observations are presented. Qualitative questions i.e., soliciting information about policies and procedures have been summarized narratively. Data analysis was conducted using StataSE 15 (StataCorp, College Station, TX, USA).

## Results

Prior to the program, out of the 177 facilities, 29 reported that cervical cancer screening was offered including but not limited to VIA, Visual inspection with Lugol's iodine (VILI), HPV DNA testing, pap smear, liquid based cytology (LBC). Of these, a total of 18 facilities offered VIA and/or HPV DNA testing. 7 facilities also offered colposcopy. The program successfully activated VIA screening and/or HPV DNA testing in an additional 159 facilities bringing the total to 177 facilities offering both VIA and HPV DNA testing. The program also supplied 43 colposcopes activating an additional 36 facilities bringing the total to 43 facilities offering colposcopy.

Between January 1, 2021, and December 31, 2021, in the 177 government health facilities 114,253 women were screened for cervical cancer; 9,064 (8%) did not consent to participate and were not included in the study. Upon further review of the 105,189 women who consented to participate, 3,318 (3%) were excluded either because there was an incomplete screening, or the observation was deemed to be a duplicate. We further excluded 18,278 (17%) women as they were outside of the program target age group, or their primary screen method was not clearly defined as VIA or HPV DNA testing. For the purposes of this analysis, we included the remaining 83,593 women.

Just over 70% of the 83,593 women were HIV negative (*n* = 59,199), 22% had an unknown HIV status (*n* = 18,351) and 7% were HIV positive (*n* = 6,043). Of the women screened, 81% received VIA as a primary screen, 19% received HPV DNA test as a primary screen, and < 1% (*n* = 49) received both at the same time.

**Fig. 2:**
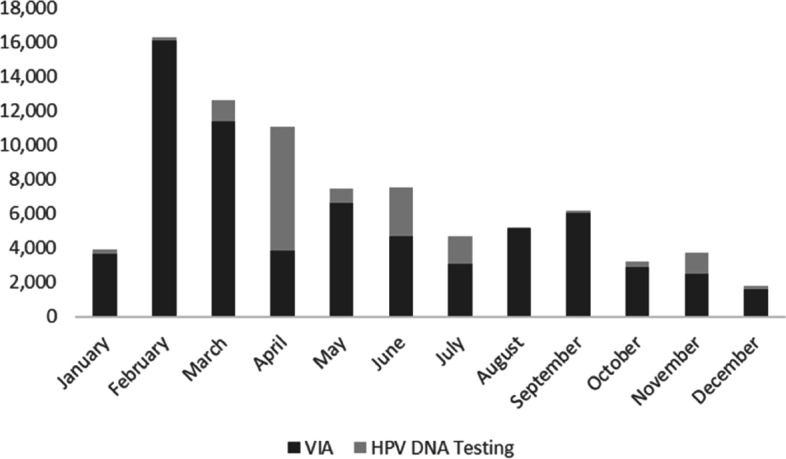
Number of VIA screens and HPV DNA tests completed by month, January – December 2021

An average of 7,000 screens were conducted each month, although total numbers varied considerably throughout the year, especially at the start of the program between January and February 2021 (Fig. 2) when uptake was highest.

The cervical cancer program supported the roll-out of 358 thermal ablation devices and 47 LEEP devices across 177 program sites. Prior to the program, out of the 177 facilities, 10 reported that treatment for precancerous lesions was offered including but not limited to cryotherapy, thermal ablation and LEEP. Of these, 1 facility offered cryotherapy only, 1 offered LEEP only, 1 offered thermal ablation and also offered cryotherapy and LEEP. The program successfully activated thermal ablation treatment in an additional 176 facilities bringing the total to 177 facilities offering thermal ablation and activated LEEP treatment in additional 40 facilities bringing the total to 43 facilities offering LEEP. Of the 67,420 women screened with VIA, 4,081 (6%) were HIV positive, VIA positivity among WLHIV was 7% compared to 2% among HIV negative women and 4% among women of unknown status (Table 1). Among the 1,912 VIA positive women 1,832 (96%) were eligible for treatment of precancerous lesions and received treatment. Nearly all (99%, *n* = 1,815) of the women treated were done on the same day following their screening and were treated using thermal ablation. The remaining were treated with LEEP (*n* = 10), cryotherapy (*n* = 6) and one woman had an unknown treatment type. Among the 190 women who were diagnosed as suspected cancer, all were referred to another facility for care and treatment and 84 (44%) had a documented referral.

**Table 1:** Cascade of care for VIA screening in Nigeria

	**HIV negative women**			**HIV positive women**			**HIV status unknown**			**All women**		
	**N**	n	%	**N**	n	%	**N**	n	%	**N**	n	%
Total tested with VIA	59199	51178	86%	6043	4081	68%	18351	12161	66%	83593	67420	81%
VIA result												
Positive, precancerous lesions	51178	1173	2%	4081	284	7%	12161	455	4%	67420	1912	3%
Negative	51178	49881	97%	4081	3,768	92%	12161	11669	96%	67420	65318	97%
Suspected of cancer	51178	124	0%	4081	29	1%	12161	37	0%	67420	190	0%
VIA positive, treatment & referral												
Received treatment	1173	1123	96%	284	270	95%	455	439	96%	1912	1832	96%
Thermal ablation	1123	1114	99%	270	267	99%	439	434	99%	1832	1815	99%
Cryotherapy	1123	3	0%	270	1	0%	439	2	0%	1832	6	0%
LEEP	1123	6	1%	270	2	1%	439	2	0%	1832	10	1%
Treatment type unknown	1123	-	0%	270	-	0%	439	1	0%	1832	1	0%
Suspected of cancer, referral												
Referred	124	124	100%	29	29	100%	37	37	100%	190	190	100%
Referred & attended referral visit	124	55	44%	29	12	41%	37	17	46%	190	84	44%
Experienced adverse event after treatment^a^	1123	5	0%	270	-	0%	439	1	0%	1832	6	0%

^a^Adverse events included heavy bleeding (*n* = 2), minor infection (*n* = 2), and unusual severe pain (*n* = 2)

Twelve thousand of the HPV DNA tests were carried out on GeneXpert platforms, with the remaining carried out on Roche Cobas platforms. The turnaround time, defined as the time interval between the specimens received in the laboratory to the time results available, ranged between approximately 9 – 76 days for the Cobas platform and in one of the GeneXpert labs, approximately 5 – 67 days. Of the 16,222 women screened using HPV DNA testing, 1,983 (12%) were HIV positive. HPV prevalence was 16% among WLHIV, 8% among HIV negative and 9% among unknown status (Table 2). In total, 310 (21%) of the 1,510 HPV positive women completed a triage examination. Following the triage exam, 44 women were deemed eligible for treatment of precancerous lesions and 42 (95%) of these women received treatment. Of the 42 women receiving treatment, 40 (95%) were treated with thermal ablation and two (5%) with LEEP. Four women were diagnosed as suspected cancer at the triage examination. All four were referred to another facility for care and treatment, only one out of the four had a documentation of attending the referral i.e., 25% referral completion rate. Triage suspected cancer, ﻿referralc

**Table 2:** Cascade of care for HPV screening in Nigeria

	**HIV negative women**			**HIV positive women**			**HIV status unknown**			**All women**		
	**N**	n	%	**N**	n	%	**N**	n	%	**N**	n	%
Total tested with HPV	59199	8042	14%	6043	1983	33%	18351	6197	34%	83593	16222	19%
HPV result												
Positive	8042	623	8%	1983	322	16%	6197	565	9%	16222	1510	9%
Negative	8042	7419	92%	1983	1661	84%	6197	5632	91%	16222	14712	91%
Triage exam^a^												
Yes	623	175	28%	322	27	8%	565	108	19%	1510	310	21%
No	623	448	72%	322	295	91%	565	457	81%	1510	1200	79%
Result of triage exam												
Negative	175	156	89%	27	23	85%	108	81	75%	310	260	84%
Positive, precancerous lesions	175	15	9%	27	3	11%	108	26	24%	310	44	14%
Suspected of cancer	175	3	2%	27	-	0%	108	1	1%	310	4	1%
Result not yet available at time of study	175	1	1%	27	1	4%	108	-	0%	310	2	1%
Triage positive, treatment and referral^b^												
Received treatment	15	15	100%	3	3	100%	26	24	92%	44	42	98%
Thermal ablation	15	13	87%	3	3	100%	24	24	100%	42	40	95%
Cryotherapy	15	-	0%	3	-	0%	24	-	0%	42	-	0%
Surgery	15	-	0%	3	-	0%	24	-	0%	42	-	0%
LEEP	15	2	13%	3	-	0%	24	-	0%	42	2	5%
Other	15	-	0%	3	-	0%	24	-	0%	42	-	0%
Triage suspected cancer, referral^c^												
Referred	3	3	100%	-	-	-	1	1	100%	4	4	100%
Referred and attended referral visit	3	-	0%	-	-		1	1	100%	4	1	25%
Experienced adverse event after treatment^d^	15	1	7%	3	-	0%	24	-	0%	42	1	2%

^a^Triage exam includes confirmatory VIA screening, pap smear, or colposcopy

^b^Includes HPV positive women who attended the triage exam and upon examination were positive for precancerous lesions and eligible for treatment

^c^Includes HPV positive women who attended the triage exam and upon examination were suspected cancer

^d^Adverse event reported included minor infection (*n* = 1)

Following treatment with thermal ablation, a total of 7 cases of adverse events were reported. Two experienced heavy bleeding, three reported a minor infection, and two reported unusual severe pain.

## Discussion

This study, nested within the first large-scale, public sector driven cervical cancer secondary prevention program in Nigeria, provides evidence that low resource settings can effectively introduce same day screen and treat services for the prevention of cervical cancer. Throughout the study period nearly 84,000 women were screened for cervical cancer within the target age group. Impressively, for women who were eligible for treatment, over 95% received it. Many women, however, did not attend necessary triage examinations and/or referral visits. This program successfully expanded access to screening and treatment services by building effective delivery systems in partnership with the Ministry of Health to not only reach women with life-saving services, but also to lay the groundwork for scale-up.

Of the 83,593 women included in this study, over 80% were screened using VIA while the remaining were screened using HPV DNA testing. Most women were screened by VIA as this method requires minimal costs and resources like findings in other setting in sub-Saharan Africa such as in Zambia, where the cervical cancer prevention program has been very effective in scaling up cervical cancer screenings using VIA and integrating in government-led clinics [16]. However, while the program was able to introduce and expand HPV DNA testing to over 16,000 women by leveraging existing multiplex testing platforms and integrating HPV testing onto devices that had spare testing capacity, HPV testing faced many systemic constraints thereby limiting its uptake. Some of the challenges faced were cumbersome sample transport processes, non-prioritization of HPV DNA testing at laboratories due to workload, unavailability of reagents, unavailability of lab consumables such as tube racks, etc. resulting in slow return of results which negatively impacted on linkage of HPV positive women to care, and periodic malfunctioning of testing platforms. There is a need to strengthen sample transport mechanisms and integrate HPV testing workflows in laboratories to drive seamless HPV testing.

Of the women screened, disaggregated by HIV status, we observed a fourfold difference in the positivity rate for VIA between WLHVIV and HIV- women, and twice the positivity for HPV between WLHIV and HIV- women. Studies have shown that women infected with HIV have an increased risk of also being HPV-infected and consequently are at higher risk for cervical cancer [17, 18], similarly we see a higher prevalence of pre-cancerous lesions among WLHIV in the three states. This further necessitates a routinely available secondary prevention program to ensure timely identification and diagnosis, and treatment while reducing the barriers to access and loss to follow up. The low proportion of total women screened who were WLHIV (only 7%) highlights a limitation of the program in reaching this population where prevalence is higher. With the primary strategy to reach WLHIV through ART clinics, we suspect the low proportion of WLHIV screened is in large part due to the COVID-19 global pandemic and procedures put into place at ART clinics to reduce risk of exposure. At the onset of the COVID-19 pandemic and ensuing lockdowns in the country, the HIV program implemented the Differentiated Service Delivery (DSD) model which resulted in people living with HIV (PLHIV) needing to visit the health facilities once every six months for drug refills as opposed to the once in two months routine prior to the pandemic. In most cases, PLHIV were even encouraged to visit community pharmacies or receive home deliveries to avoid visits to the clinic. This reduced the total population of WLHIV attending ART clinics thereby impacting screening numbers. To reach this subpopulation, the program has since conducted community-based outreaches targeted at WLHIV and worked closely with PEPFAR implementation partners to leverage on their network and structures such as engaging directly with mentor mothers in ART clinics to reach WLHIV. The screening coverage achieved by the program in one year is attributable in some part to the successful demand generation strategies which were employed such as the collaboration with CSOs and engagement of community mobilisers. Due to the adaptive and agile demand generation strategies employed in the program, there was increased awareness of the screening services and subsequently increased uptake. A successful integrated service delivery model for cervical cancer secondary prevention in public health settings requires strong community participation and targeted multi-dimensional demand generation approaches.

One key observation from this study was the testing trends. As shown in Fig. 2, there were fluctuations in the testing numbers with declines observed from March 2021. Critical challenges to the effective delivery of services in the facilities often led to the inability of the system to fully meet demand for screening and treatment. Insufficient health workers led to increased workload with the added screen and treat program, significant sample transportation delays, and lack of consumables at facilities and testing laboratories affected treatment and both HPV and VIA screening. This highlights the importance of strengthening the health system to sustainably introduce and integrate secondary prevention services for cervical cancer as part of routine facility services in the public sector in Nigeria.

We believe that the high rates of treatment observed in this study can be attributed to a same day screen and treat approach as there are reduced chances of loss to follow-up, which positively impacts cervical cancer control [19]. Thermal ablation as a treatment method was highly accepted by HCWs. Previously treatment of precancerous lesions was typically carried out by doctors. However, with the program training less specialized HCWs i.e., nurses and community health extension workers, this increased client’s access to care. Acceptance could also be linked to the ease it provided for HCWs to carry out treatment to eligible clients, with very little pain, side effects and quicker recovery process for clients. HCWs also noted it was easier to manage and disinfect/clean thermal ablation devices after every use. The program initially had challenges with low confidence for thermal ablation among health workers, thus, conducted refresher trainings, introduced regular HCW review meetings and added clinical mentoring to support health workers develop the confidence to practice their newly acquired skills and training. This occurrence demonstrated the need for continued on-the-job training, and provider support to sustain service provision and program quality.

This study had some limitations. Poor linkage to care for women who received HPV DNA testing and high rates of loss to follow up was observed, due to the triage requirement in the clinical protocol. Nearly all women screened using an HPV DNA test who were deemed eligible for treatment received treatment; however, this represents only a small proportion (3%) of the women who were HPV-positive upon the initial screen. Tracking patients throughout the continuum of care proved challenging leading to the inability to follow-up with clients who needed further screening. Another limitation is that the accuracy and completeness of the study data are questionable given that the primary source of data, standardized medical records were completed routinely by health care providers, and not study specific data collectors. Steps were taken though to ensure a higher quality of data such as training health care providers on how to complete the registers and chasing down missing information after the fact. These findings are limited to women who were already seeking care at the focal health facilities and accepted cervical cancer screening services. The health facilities have geographic representation across all three states and efforts to implement demand generation activities were undertaken throughout program implementation; however, these results may miss those who did not seek care at public health facilities or those that refused to be screened. The timing of data collection may have implications on triage follow-up and referral completion rates, given that some women had a longer time to return for these services than others. Finally, challenges in the procurement of HPV test kits resulting in delayed arrival of test kits in country, affected the rate of HPV screening. However, steps are being taken to put in place a more sustainable means to provide HPV testing in country.

## Conclusions

Through the Cervical Cancer Secondary Prevention Program, women are able to access cervical cancer screening and treatment of precancerous lesions within public health facilities. As at December 2022, over 200,000 women have accessed effective secondary prevention services using VIA and/or HPV DNA testing. The program has yielded several successes which can be attributed to the effective delivery systems put into place at the start of the program, such as leveraging existing multiplex diagnostic platforms to expand HPV DNA testing, integration of cervical cancer screening into existing, routine health services targeting women of reproductive age, successful demand generation and same day screen and treat where possible. Despite the program's successes, there was apparent low linkage to care particularly among those screened using HPV DNA testing; suggesting a need to improve the mechanisms in place to track patients across the continuum of care. VIA remains the most viable model, HPV DNA testing is also a promising model for cervical cancer screening programs in LMICs. Implementation studies should be conducted to provide insights to improve the utilization of the existing centralized and point of care (POC) platforms to facilitate same day results. Additional efforts into strengthening linkage and follow-up systems are also necessary for seamless HPV DNA testing.

## Data Availability

The datasets used and/or analyzed during the current study are available from the corresponding author on reasonable request.

## Abbreviations

ART: Antiretroviral therapy
CHAI: Clinton Health Access Initiative
CHEW: Community health extension workers
CSO: Civil Society Organizations
DNA: Deoxyribonucleic acid
HCW: Healthcare worker
HPV: Human papillomavirus
LEEP: Loop Electrosurgical Excision Procedure
LMIC: Low- and middle-income countries
NHREC: National Health Research Ethics Committee
PEPFAR: U.S. President’s Emergency Plan for AIDS Relief
SCJ: Squamocolumnar junction
THF: Tertiary health facilities
VIA: Visual inspection with acetic acid
WHO: World Health Organization
WLHIV: Women living with HIV

## References

[1] Arbyn M, Weiderpass E, Bruni L, de Sanjosé S, Saraiya M, Ferlay J (2020). Estimates of incidence and mortality of cervical cancer in 2018: a worldwide analysis. Lancet Glob Health.

[2] Nigeria Fact Sheet 2020. Available from: https://gco.iarc.fr/today/data/factsheets/populations/566-nigeria-fact-sheets.pdf. [Cited 2023 Jan 10].

[3] Vaccarella S, Franceschi S, Zaridze D, Poljak M, Veerus P, Plummer M (2016). Preventable fractions of cervical cancer via effective screening in six Baltic, central, and eastern European countries 2017–40: a population-based study. Lancet Oncol.

[4] Schiffman M, Castle PE, Jeronimo J, Rodriguez AC, Wacholder S (2007). Human papillomavirus and cervical cancer. Lancet Lond Engl.

[5] Genital HPV Infection – Basic Fact Sheet. 2022. Available from: https://www.cdc.gov/std/hpv/stdfact-hpv.htm. [Cited 2023 Jan 12].

[6] World Health Organization. Comprehensive cervical cancer control: a guide to essential practice [Internet]. 2nd ed. Geneva: World Health Organization; 2014. p. 364. Available from: https://apps.who.int/iris/handle/10665/144785. [Cited 2023 Jan 12]. 25642554

[7] World Health Organization. Global strategy to accelerate the elimination of cervical cancer as a public health problem. World Health Organization; 2020. p. 52. Available from: https://apps.who.int/iris/handle/10665/336583. [Cited 2023 Jan 12].

[8] Denny LA, Sankaranarayanan R, De Vuyst H, Kim JJ, Adefuye PO, Alemany L (2013). Recommendations for cervical cancer prevention in sub-saharan Africa. Vaccine.

[9] Vedantham H, Silver MI, Kalpana B, Rekha C, Karuna BP, Vidyadhari K (2010). Determinants of VIA (Visual Inspection of the Cervix After Acetic Acid Application) Positivity in Cervical Cancer Screening of Women in a Peri-Urban Area in Andhra Pradesh, India. Cancer Epidemiol Biomark Prev Publ Am Assoc Cancer Res Cosponsored Am Soc Prev Oncol.

[10] Mustafa M, Jindal A, Singh P (2010). Visual inspection using acetic acid for cervical cancer in low resource settings. Med J Armed Forces India.

[11] WHO guidelines for screening and treatment of precancerous lesions for cervical cancer prevention. Supplemental material: GRADE evidence-to-recommendation tables and evidence profiles for each recommendation. Available from: https://apps.who.int/iris/bitstream/handle/10665/96735/WHO_RHR_13.21_eng.pdf?sequence=1. [Cited 2023 Jan 12].

[12] World Health Organization - 2013 - WHO guidelines for screening and treatment of prec.pdf. Available from: https://apps.who.int/iris/bitstream/handle/10665/94830/9789241548694_eng.pdf. [Cited 2023 Jan 12]. 24716265

[13] Dolman L, Sauvaget C, Muwonge R, Sankaranarayanan R (2014). Meta-analysis of the efficacy of cold coagulation as a treatment method for cervical intraepithelial neoplasia: a systematic review. BJOG Int J Obstet Gynaecol.

[14] Efficacy, Safety, and Acceptability of Thermal Coagulation to Treat Cervical Intraepithelial Neoplasia: Pooled Data From Bangladesh, Brazil and India | Nessa | Journal of Clinical Gynecology and Obstetrics. Available from: https://www.jcgo.org/index.php/jcgo/article/view/464. [Cited 2023 Jan 12].

[15] Use of thermo‐coagulation as an alternative treatment modality in a ‘screen‐and‐treat’ programme of cervical screening in rural Malawi - PMC. Available from: https://www.ncbi.nlm.nih.gov/pmc/articles/PMC5084797/. [Cited 2023 Jan 12]. 10.1002/ijc.30101 PMC5084797 27006131

[16] Pry JM, Manasyan A, Kapambwe S, Taghavi K, Duran-Frigola M, Mwanahamuntu M (2021). Cervical cancer screening outcomes in Zambia, 2010–19: a cohort study. Lancet Glob Health.

[17] Vernon SD, Holmes KK, Reeves WC (1995). Human papillomavirus infection and associated disease in persons infected with human immunodeficiency virus. Clin Infect Dis Off Publ Infect Dis Soc Am.

[18] Schulz TF, Boshoff CH, Weiss RA (1996). HIV infection and neoplasia. The Lancet.

[19] Shahnaz S, Hira HM, Begum KN, Akhter R, Sharmin S (2021). “Screen and Treat” approach among via positive women during cervical cancer screening program: experience at low resource setting. Mymensingh Med J MMJ.

